# 

**Published:** 1930

**Authors:** 


					?
? > : r.'?
v?i YI.VTT- No. 178.
if wV-.?j -1 ,
?g^ga^raaHP'sgMwa
??*' A*!
. v->' : -
WINTER. 1930.
"V ,f=w . ' ?
: .V +>\ # JT, ^
ico-1
_ - - -----
PtTBLISHBD UNDKU THE AUSPICES OF
THE BRISTOL MEDICO-CH!IRURGICAJL SO
I: %S'< . k'-#'#-'
J. A. NIX(
WITH WHOM ARB ASSOCIATED
E. W. HEY GROVES, M.S., F.R.O.S., B.Sc.
E. WATSON-WILLIAMS, M.O., Oh.M., F.R.O.S.E.,
iy
Editor.
Editorial Secretary: A. L. FLEMMING, M.B., Ch.B.
>W>" .
??. ?? "?   *?      "? ?" ,   "?"?
: ' ? ' ? -
Price Three Shillings net.

				

